# The secretome of *Staphylococcus aureus* strains with opposite within-herd epidemiological behavior affects bovine mononuclear cell response

**DOI:** 10.1186/s13567-023-01247-w

**Published:** 2023-12-14

**Authors:** Susanna Di Mauro, Joel Filipe, Alessia Facchin, Laura Roveri, Maria Filippa Addis, Valentina Monistero, Renata Piccinini, Giulia Sala, Davide Pravettoni, Clarissa Zamboni, Fabrizio Ceciliani, Cristina Lecchi

**Affiliations:** 1 Department of Veterinary Medicine and Animal Science, Università degli Studi di Milano, via dell’Università 6, 26900 Lodi, Italy; 2 Laboratorio di Malattie Infettive degli Animali–MILab, Università degli Studi di Milano, Via dell’Università 6, 26900 Lodi, Italy; 3 Department of Veterinary Sciences, University of Pisa, via Livornese s.n.c, 56122 San Piero a Grado, Italy

**Keywords:** *Staphylococcus aureus*, secretome, PBMC polarization, immune response, cow

## Abstract

*Staphylococcus aureus* modulates the host immune response directly by interacting with the immune cells or indirectly by secreting molecules (secretome). Relevant differences in virulence mechanisms have been reported for the secretome produced by different *S. aureus* strains. The present study investigated the *S. aureus* secretome impact on peripheral bovine mononuclear cells (PBMCs) by comparing two *S. aureus* strains with opposite epidemiological behavior, the genotype B (GTB)/sequence type (ST) 8, associated with a high within-herd prevalence, and GTS/ST398, associated with a low within-herd prevalence. PBMCs were incubated with different concentrations (0%, 0.5%, 1%, and 2.5%) of GTB/ST8 and GTS/ST398 secretome for 18 and 48 h, and the viability was assessed. The mRNA levels of pro- (IL1-β and STAT1) and anti-inflammatory (IL-10, STAT6, and TGF-β) genes, and the amount of pro- (miR-155-5p and miR-125b-5p) and anti-inflammatory (miR-146a and miR-145) miRNAs were quantified by RT-qPCR. Results showed that incubation with 2.5% of GTB/ST8 secretome increased the viability of cells. In contrast, incubation with the GTS/ST398 secretome strongly decreased cell viability, preventing any further assays. The GTB/ST8 secretome promoted PBMC polarization towards the pro-inflammatory phenotype inducing the overexpression of IL1-β, STAT1 and miR-155-5p, while the expression of genes involved in the anti-inflammatory response was not affected. In conclusion, the challenge of PBMC to the GTS/ST398 secretome strongly impaired cell viability, while exposure to the GTB/ST8 secretome increased cell viability and enhanced a pro-inflammatory response, further highlighting the different effects exerted on host cells by *S. aureus* strains with epidemiologically divergent behaviors.

## Introduction

Mastitis is a primary health and economic issue addressed in dairy farming. Among the numerous mastitis-causing pathogens, *Staphylococcus aureus* is one of the most relevant worldwide. It can establish both acute and chronic infections often leading to subclinical mastitis. Due to its ability to persist inside the mammary gland [1, 2] and to internalize within mammary epithelial cells and phagocytes such as monocytes, *S. aureus* can evade the host immune response [3, 4]. The immune escape strategies vary greatly in vivo and in vitro, according to the bacterial genotype [5–8]. *S. aureus* genotypes can have strikingly different genomic, transcriptomic, and proteomic profiles, as well as diverse pathogenic and epidemiological behaviors [9–12]. In many European countries, most *S. aureus* strains isolated from cows with intramammary infection (IMI) belong to genotype B (GTB), generally corresponding to Sequence Type (ST) 8 [13], which is a highly contagious bovine-adapted strain [14, 15]. Genotype S (GTS), corresponding to ST398, is more likely associated with sporadic IMI and can affect livestock animals and humans, developing antimicrobial resistance and, thus, representing a public health issue [16].

Secreted molecules are essential elements in bacterial infections. Based on the released virulence factors, they can exert different activities, killing target cells or helping the bacterial pathogen establishment in the host cell [17]. A recent comparative study provided a thorough characterization of the secreted proteins (secretome) of GTB/ST8 and GTS/ST398, connecting their secretome profiles with the respective epidemiological behaviors: GTB/ST8 preferentially released virulence factors associated with the infection development and persistence, avoiding both the innate and adaptive humoral responses, while GTS/ST398 secretomes enhanced cellular damage and inflammation [10]. The same study demonstrated that the secretome of GTB/ST8 did not exert cytotoxic activities on bovine PBMCs, further supporting the hypothesis of its ability to evade the host immune response. At the same time, GTS/ST398 reduced cell viability at high concentrations (2.5 and 10%) [10]. Although the *S. aureus* ability to evade the host immune response by directly interacting with the immune cells has been previously investigated [7], no data on the immunomodulatory ability of secreted molecules (secretome) has been reported on bovine immune cells so far. This study aimed to investigate whether GTB/ST8 and GTS/ST398 secretomes in vitro could modulate the bovine immune response of peripheral blood mononuclear cells at the molecular level, focusing on genes and miRNAs related to the M1/Th1 and M2/Th2 phenotypes polarization.

## Materials and methods

### Purification of PBMC from bovine peripheral blood

Peripheral blood from clinically healthy multiparous Holstein cows at the second parity in their second trimester (90 to 180 DIM) of lactation was collected in sterile tubes treated with K_2_EDTA (Vacutainer) during routine slaughtering procedures. The isolation of PBMC was performed using Ficoll-Paque Plus (GE Healthcare Bio-Sciences AB, Uppsala, Sweden) 1.077 g/mL density gradient centrifugation, as previously described [18]. Briefly, whole blood was centrifuged at 1260 × *g* for 30 min at 18 °C to collect the buffy coat. The buffy coat was diluted 1:5 with cold PBS + EDTA 2 mM without Ca^2+^ and Mg^2+^ (Sigma-Aldrich, St. Louis, MO, USA), layered on Ficoll, and centrifuged at 1700 × *g* for 30 min at 4 °C to isolate PBMC ring. The PBMC ring was collected and washed twice with cold PBS + EDTA 2 mM by centrifuging at 500 × *g* for 7 min at 4 °C to remove the platelets. The pellet was treated with Red Blood Lysis Buffer (Sigma-Aldrich) and then washed with cold PBS + EDTA 2 mM by centrifuging at 500 × *g* for 7 min at 4 °C to remove red blood cells. Isolated PBMC were counted using an Automatic Cell Counter (BioRad) and resuspended at the desired final concentration in RPMI 1640 medium with 25mM Hepes and l-glutamine, complemented with 1% nonessential amino acid solution (100×), 1% penicillin-streptomycin solution (100×; Euroclone, Milano, Italy), and 10% fetal bovine serum (FBS; Sigma-Aldrich).

### Preparation and quantification of the *S. aureus* secretome

The stock solution of *S. aureus* secretomes concentrated at 8–10 µg/µL was produced as previously reported [10]. Briefly, bacteria were revitalized in Brain Heart Infusion (BHI) broth overnight at 37 °C. Overnight culture suspensions were then diluted 1:100 in RPMI-1640 and incubated at 37 °C with agitation for 3.5 h. The bacterial culture was centrifuged at 9300 × *g* for 5 min, and proteins were processed with Amicon Ultra-0.5 centrifugal filter units with Ultracel-10 membrane (Millipore, Billerica, MA, USA). Protein concentration was assessed using the Pierce™ 660 nm Protein Assay Kit (Thermo Scientific, San Jose, CA, USA).

### Cell viability assay

Cell viability was determined using Cell Proliferation Kit, I (MTT) from Roche, following the manufacturer’s instructions. PBMC from 6 different animals (1 × 10^5^ cells/well) were challenged with increasing concentrations (0.5%, 1%, 2.5%) of GTB/ST8 and GTS/ST398 secretomes in 96-well plates, incubated at 37 °C and 5% CO_2_ for 18 and 48 h. The secretome concentrations were selected based on previously reported results [10]. Cells without secretome were included as a control. After incubation, the MTT reagent (10 µL) was added and incubated for 4 h at 37 °C. Solubilization buffer (100 µL) was then added and incubated overnight. The absorbance was measured with a Lab Systems Multiskan plate reader spectrophotometer (Lab, Midland, Canada) at 550 nm.

### PBMC stimulation with *S. aureus* secretome

A total of 5 × 10^5^ cells/well from 10 animals were seeded in triplicate in sterile 24-well plates (Falcon COD 351147) and incubated for 18 and 48 h at 37 °C and 5% CO_2_ with increasing concentrations of *S. aureus* secretome (0.5%, 1%, and 2.5%). Cells without secretome were included as a control. After incubation, the cells were washed with PBS and centrifuged at 500 × *g* for 7 min. Finally, PBMCs were lysed, adding 700 µL of Fenozol plus (A&A biotechnology COD 203-50P), and stored at −80 °C.

### Long and small RNA extraction and quantification

Long and small RNAs were extracted from cells using a MicroRNA concentrator kit (A&A biotechnology COD 010AAB035), following the manufacturer’s instructions. *Caenorhabditis elegans* miRNA cel-miR-39 (25 fmol final concentration) was added and used as exogenous synthetic spike-in control. RNA concentration and quality were assessed using a NanoDrop ND-1000 UV–vis spectrophotometer (NanoDrop Technologies Inc., Wilmington, DE, USA). The Minimum Information for Publication of Quantitative Real-Time PCR (MIQE) guidelines were followed [19].

### mRNA quantification

The reverse transcription (RT) reaction from 190 ng mRNA was carried out using the iScript cDNA Synthesis Kit (BioRad), according to the manufacturer’s instructions. The expression of genes involved in the Th1-M1 and Th2-M2 pathways was quantified using qPCR, and the reaction was carried out in duplicate. The selected targets included pro- (IL1-β and STAT1) and anti-inflammatory (IL-10, STAT6, and TGF-β) genes, amplified using previously described primers [20]. The reaction was carried out in a scaled-down reaction volume (15 µL) in a CFX Connect Real-Time PCR Detection System (BioRad), using 7.5 µL of SsoFast™ EvaGreenSupermix (Bio-Rad, California, USA), forward and reverse primers, 1 µL of cDNA sample, and RNase- and DNase free water to make up the remaining volume. The thermal profile consisted of 50 °C for 2 min, 95 °C for 3 min, 40 cycles of 95 °C for 15 s, and 60 °C for 30 s. Two reference genes (YWHAZ and H3F3A) were selected [20], and their mean was used for normalization using the 2^−ΔΔCq^ method.

### miRNA quantification

To synthesize cDNA from the isolated small RNA, the TaqMan® Advanced miRNA Assays kit (Thermo Fisher Scientific, A25576) was used according to the manufacturer’s recommendations.

MicroRNAs were selected according to previous studies in which these miRNAs were found to exert pro- (miR-155-5p and miR-125b-5p) and anti-inflammatory (miR-146a and miR-145) activities [21–23]. The selected miRNAs included miR-155-5p (assay ID 477927_mir), miR-125b-5p (assay ID 480907_mir), miR-145-5p (assay ID 480938_mir), and miR-146a-5p (assay ID 478399_mir). The reaction was carried out in a scaled-down reaction volume (15 µL) in a CFX Connect Real-Time PCR Detection System (BioRad), using 7.5 µL of Advanced Master Mix 2X (Thermo Fisher Scientific, 4444557), 0.75 µL of miRNA-specific TaqMan advance assay reagents (20×), 1 µL of cDNA sample, and RNase-free water to reach the final volume. Each sample was tested in duplicate. The thermal cycling profile of the reaction was: 50 °C for 2 min, 95 °C for 3 min, 40 cycled at 95 °C for 15 s, and 60 °C for 30 s. To evaluate the stability of reference miRNAs, namely miR-320a-3p (assay ID 478594_mir) and miR-187-5p (assay ID 477941_mir), a geNorm analysis was performed using Biogazelle’s qbase+ software. Data normalization was carried out using the arithmetic mean of two reference miRNAs. Relative quantification of each miRNA was calculated using the BioRad CFX Maestro Software by the 2^−ΔΔCq^ method.

### Statistical analysis

Statistical analysis was carried out using GraphPad Prism 9.0.0 (San Diego, CA, USA). For the viability assay, the normal distribution of the data was assessed by performing the Shapiro-Wilk test. Not normally distributed data were analyzed using Friedman and Dunn’s multiple comparison tests. A grouped analysis was performed using multiple comparisons 2-way ANOVA test to analyze the mRNA and miRNA expression.

## Results

### The *S. aureus* secretomes had opposite effects on cell viability

**Figure 1:**
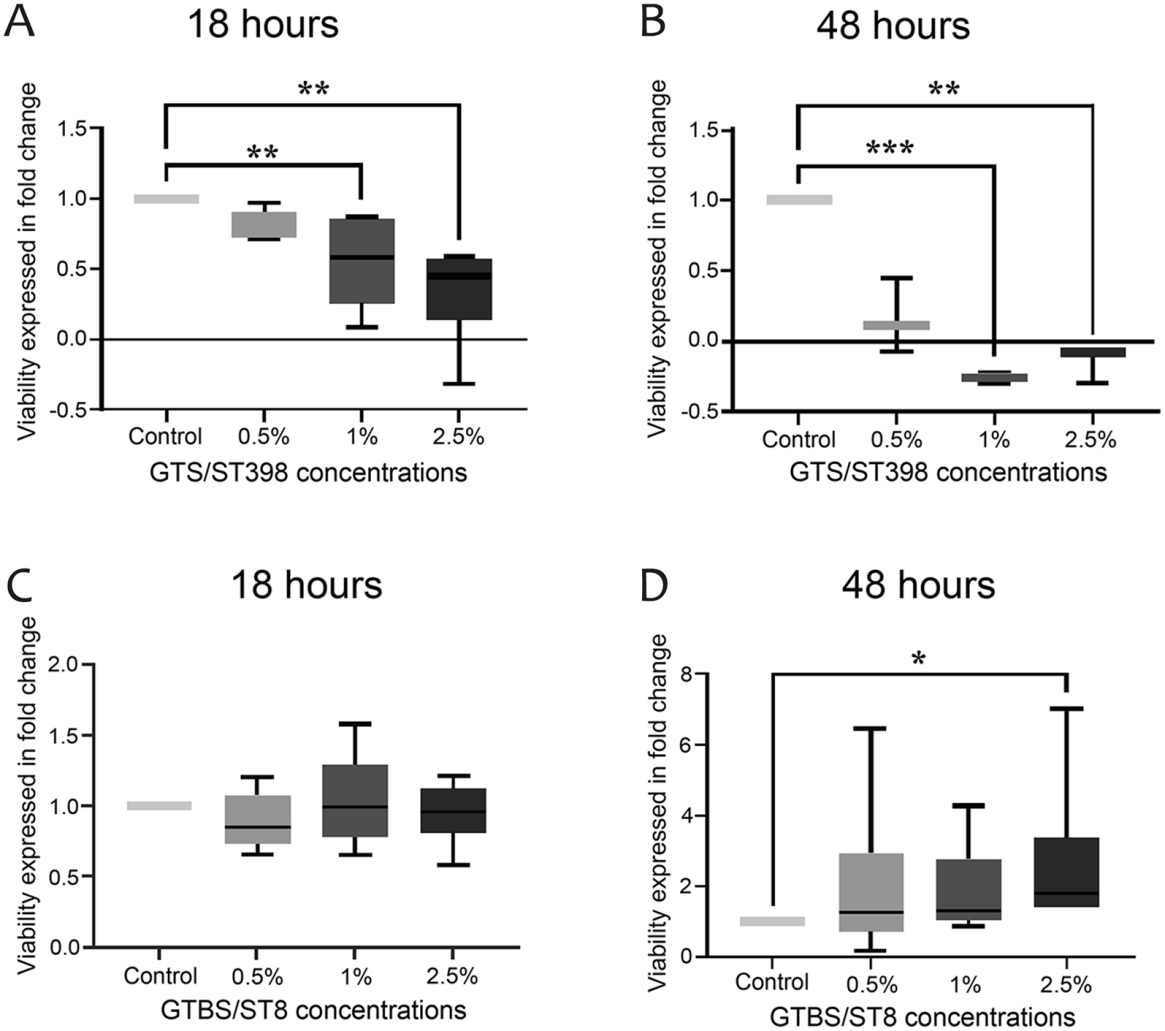
**PBMC viability after (A–C) 18- and (B–D) 48-h incubation with increasing concentration of GTS/ST398 and GTB/ST8 secretome, respectively.** The viability is expressed as fold-change compared to the control (cells incubated without secretome) in six biological replicates. Significance was accepted at *P <* 0.05 (*), *P* < 0.01 (**), and *P <* 0.001 (***). The lines inside the boxes denote the median.

After 18 h of incubation with GTS/ST398-secreted molecules (Figure 1A), PBMC treated with 1% and 2.5% concentration decreased their viability by 0.7 and 0.48 folds compared with the control (*P* = 0.0052 and *P* = 0.0010, respectively). After 48 h of incubation (Figure 1B), 1% and 2.5% concentrations significantly decreased immune cell viability by 0.25 and 0.08 folds compared with the control (*P* = 0.0006 and *P* = 0.0086, respectively). On the other hand, no effect on viability was observed on cells incubated with GTB/ST8 secretome for 18 h (Figure 1C); indeed, after 48 h of incubation, the highest concentration of secretome (2.5%) increased the PBMC viability by 1.4-folds compared with the control (*P* = 0.011; Figure 1D). Since cell viability results showed that GTS/ST398-secreted molecules have a strong cytotoxic effect, further molecular assessments were carried out testing only the GTB/ST8 secretome.

### The GTB/ST8 secretome increased the expression level of proinflammatory genes

**Figure 2:**
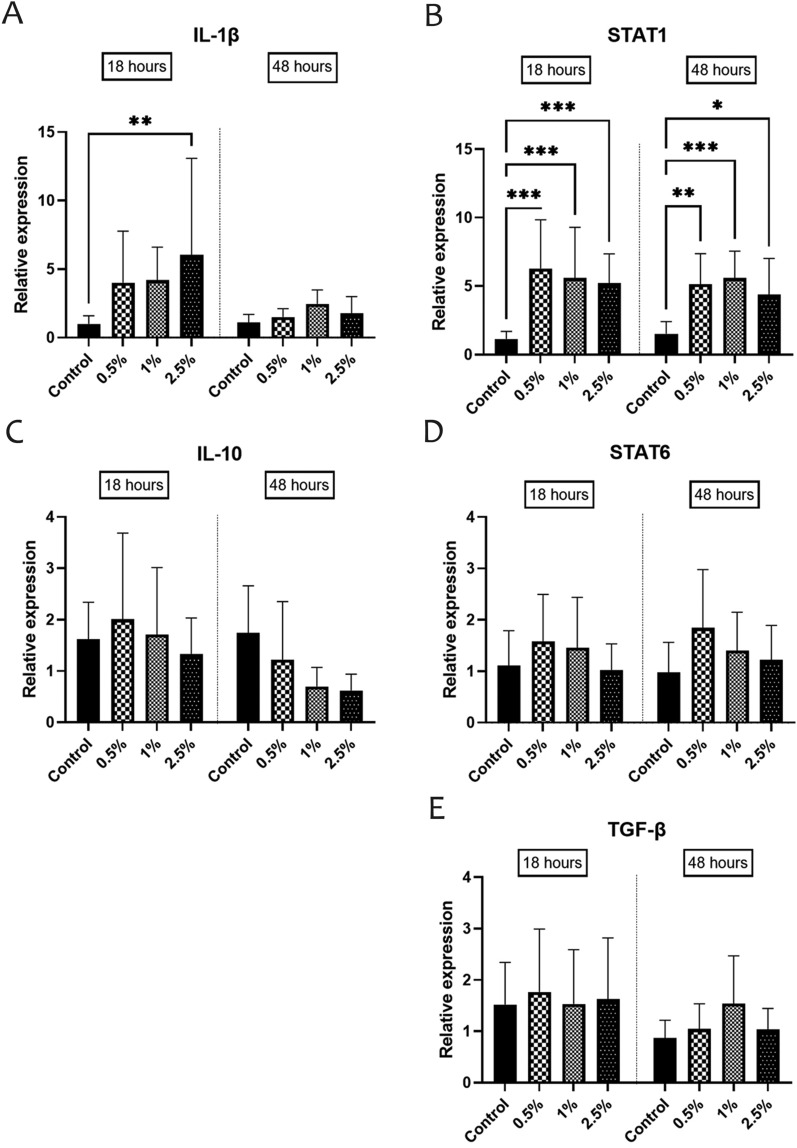
**Relative expression of mRNA related to M1/Th1 phenotype (A and B) and M2/Th2 phenotype (C, D, and E) in PBMC incubated for 18 and 48 h with increasing concentrations of GTB/ST8 secreted proteins.** Cells incubated without secretome were used as control. Data are means ± SD of 10 different animals. Significance was accepted for *P* < 0.05 (*), *P* < 0.01 (**) and *P* < 0.001 (***).

To evaluate the ability of the GTB/ST8 secretome to promote a pro- or anti-inflammatory gene expression at the molecular level, PBMCs were incubated for 18 and 48 h with increasing concentrations (0.5%, 1%, and 2.5%). The results are presented in Figure 2.

The expression of IL1β was upregulated after the incubation with 2.5% secretome for 18 h (IL1β_FC(2.5%/control)_ = 6.1, *P* = 0.014), while no effect was observed after 48 h (Figure 2A). STAT1 abundance was significantly affected by the challenge with GTB/ST8-secreted molecules. After 18 h, cells treated with 0.5%, 1% and 2.5% secretome increased the expression of STAT1 (STAT1_FC(0.5%/control)_ = 5.5, *P* = 0.001; STAT1_FC(1%/control)_ = 4.9, *P* = 0.003; STAT1_FC(2.5%/control)_ = 4.6, *P* = 0.009, respectively; Figure 2B). After 48 h, cells stimulated with 0.5%, 1% and 2.5% secretome upregulated the expression of STAT1 compared with the control (STAT1_FC(0.5%/control)_, *P* = 0.008, STAT1_FC(1%/control)_, *P* = 0.001, STAT1_FC(2.5%/control)_, *P* = 0.029, respectively; Figure 2B).

The challenge with GTB/ST8 secretome did not affect the expression of anti-inflammatory genes (Figures 2C–E).

### The GTB/ST8 secretome increased the expression level of proinflammatory miR-155-5p

**Figure 3:**
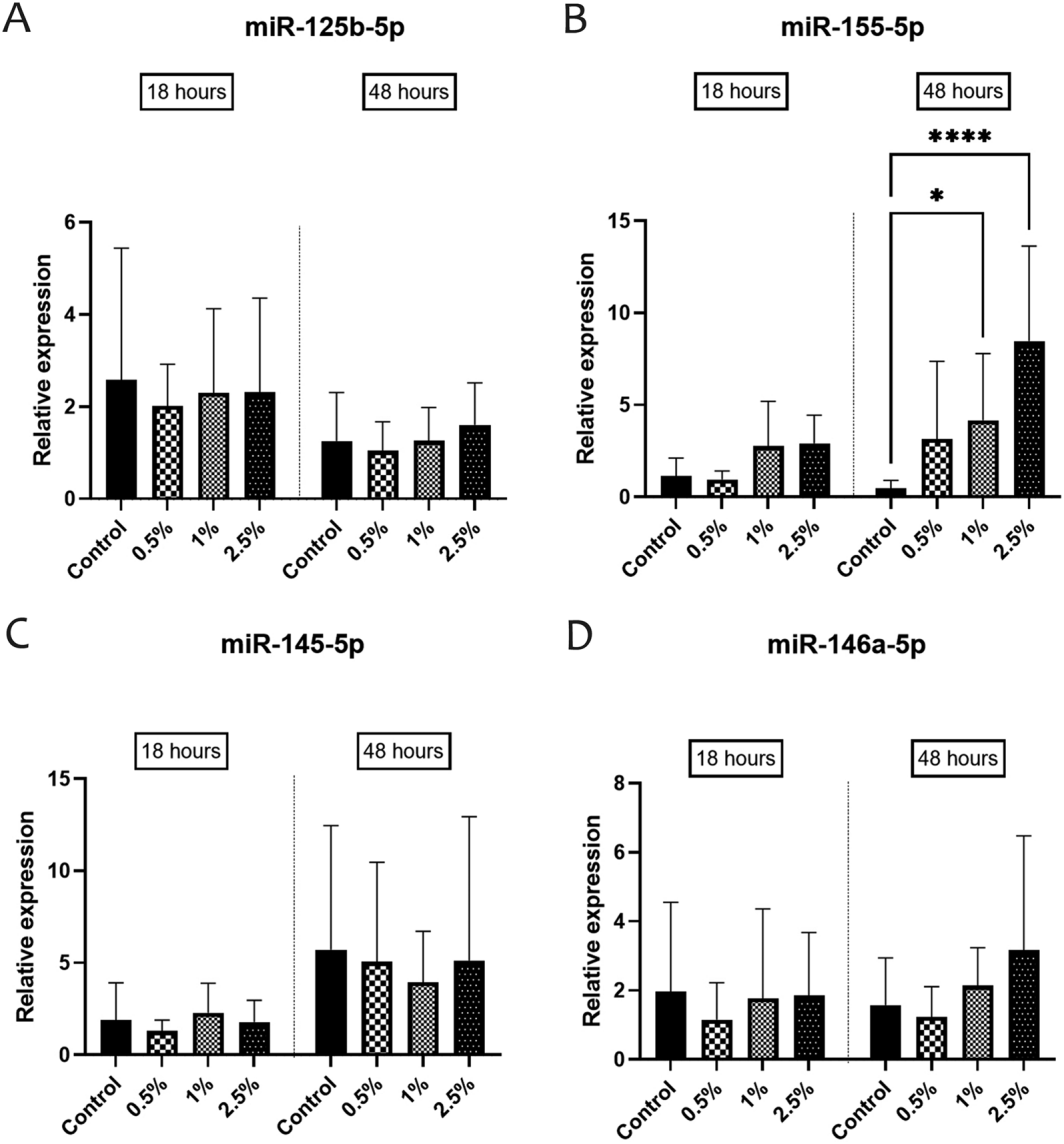
**Relative expression of miRNA related to M1/Th1 phenotype (A and B) and M2/Th2 phenotype (C and D), in PBMC, incubated for 18 and 48 h with increasing concentrations of GTB/ST8 secreted proteins (0.5%, 1%, and 2.5%).** Cells incubated with medium only were used as control. Data are means ± SD of 10 different animals. Significance was accepted for *P* < 0.05 (*), *P* < 0.01 (**), *P* < 0.0001 (****).

The miRNA’s relative abundance was quantified using RT-qPCR. Analysis of the reference miRNAs expression stability by geNorm indicated that both were suitable with average M values of 0.787. Their mean was used for the normalization of the relative quantification data experiments (Figure 3).

The relative abundance of miRNA targets involved in modulating pro- and anti-inflammatory responses was evaluated. All targets were quantifiable in the samples at different time points, and one of the four tested targets was differentially expressed. In detail, the level of miR-155-5p increased after 48 h of challenge with 1% and 2.5% GTB/ST8 compared with the control (miR-155-5p_FC(1%/control)_ = 8.87, *P* = 0.014; miR-155-5p _FC(2.5%/control)_ = 18, *P* < 0.0001) (Figure 3B).

## Discussion

In this study, we aimed to assess the ability of the secretome of *S. aureus* strains with opposite epidemiological behavior to micromanage the bovine immune response in vitro by acting differentially on PBMC activation.

*Staphylococcus aureus* can express a wide array of secreted virulence factors, which can interact with innate and adaptive immune responses, influencing leukocyte activation [24, 25], and the particular virulence pattern of each strain is strongly related to the *S. aureus* genotype [8, 12, 14, 26]. Recently, a comparative secretome study has analyzed the secreted protein profile of GTB/ST8 and GTS/ST398 strains [10]. Specific proteins, such as immunoglobulin G binding protein A (Spa), immunoglobulin-binding protein (Sbi), and the staphylococcal complement inhibitor (Scin), were found among the differentially secreted proteins of these two genotypes and were identified as promoters of host immune evasion by acting on different pathways [10]. That study also provided the first evidence that the molecules released in culture by the two genotypes had dramatically different impacts on cell viability, showing that exposure to low concentrations of the GTS/ST398 secretomes could lead to cell death. On the other hand, following previous studies, the *S. aureus* virulence factors may manipulate the host’s immune response, alternately activating a pro- or anti-immune response [27].

Mononuclear cells are a heterogeneous cell population composed of monocytes and lymphocytes, characterized by remarkable plasticity and diversity. Both functions are settled in response to microenvironmental signals, driving the polarized programs. The polarization extremes are represented by the M1/Th1 and M2/Th2 dichotomy, which occurs in pathophysiological conditions [28]. This dynamic skewing leads the cells to exert opposed functions: the M1/Th1 phenotype is involved in the onset of inflammation, while the M2/Th2 phenotype is involved in its resolution. M1/Th1 phenotype can be activated in response to microbial stimuli enhancing the secretion of proinflammatory cytokines, including IL1-β, IL-6, and TNF-α [29, 30].

Conversely, M2/Th2 lineages are involved in angiogenesis and tissue remodeling pathways, expressing anti-inflammatory cytokines, such as TGF-β and IL-10, and contributing to the resolution of the inflammation [18, 29, 30]. The regulation and the balance between these phenotypes are crucial for the correct onset and resolution of inflammation. However, the functional heterogeneity is only partially reflected by different phenotypes and morphological appearances, while different transcriptional programs, specifically activated by microenvironmental signals, enhance functional polarization [31]. STAT signaling plays a key role in the modulation of the immune response, as STAT1 can be used as a marker of the M1 polarization while STAT6 of the M2 phenotype. STAT1 is crucial for the immune response against bacterial infection, and a decrease in its activity is linked to a higher bacterial infection susceptibility [32]. After 18 and 48 h, we observed that the incubation of PBMC with the GTB/ST8 secretome led to a significant increase in the expression of STAT1 compared to the control. The STAT1 mRNA was also significantly upregulated in cells stimulated with a low concentration of secretome. A similar effect was previously observed in human monocyte-derived macrophages stimulated with *S. aureus* [33]. This suggests the critical and multifaceted role secreted molecules played in eliciting the host immune response. The STAT1 expression can be activated by the presence of Gram-positive bacteria such as *S. aureus* thanks to the binding effect of lipoteichoic acid (LTA) on the bacterial surface with the monocyte Toll-like receptors (TLR2) [34, 35]. The present results are therefore consistent with what has been reported in the literature.

The innate immune response occurs after sensing DAMPs and PAMPs as the molecules released by *S. aureus* by immune cells promoting the transcription of pro-IL-1α/β and other cytokines via TLR/MYD88/NF-kB pathway [36, 37]. PBMCs stimulated with GTB/ST8 secretome (2.5%) for 18 h significantly increased the expression of IL-1β. This proinflammatory cytokine can increase endothelial cell permeability and stimulate the release of chemokines, summoning inflammatory cells, including neutrophils and macrophages [38]. The IL-1β exerts wide-ranging effects in modulating immune cells and plays a pivotal role in controlling *S. aureus* infection, promoting phagocytosis and killing by neutrophils and macrophages [35, 39]. Moreover, T cell expansion is promoted by IL-1β toward Th1, Th2, and Th17 [40–42]. Souza et al. [24] demonstrated that bovine PBMC stimulated with different *S. aureus* strains causing persistent IMI increased IL-17 A and IFN-γ release in the supernatant, while only *S. aureus* strain promoted the lymphocyte polarization toward CD4^+^ and CD8^+^ phenotypes. The present work demonstrated that the challenge with 2.5% secretome increased the expression of IL-1β after 18 h and the PBMC viability after 48 h, supporting the hypothesis that *S. aureus* GTB/ST8 secreted molecules may induce lymphocyte activation and proliferation in vitro, also triggering an adaptive immune response. The results are consistent with previously reported data, which demonstrated that the innate immune response, mediated by monocytes, macrophages, Natural Killer cells, and cytokines, including IL-1β, predominates in the early stage of mammary gland infection regulating the expression of adhesins by endothelial cells and neutrophil chemotaxis and then stimulating the acquired immune response [43].

The *S. aureus* secretome enhances the expression of miR-155-5p, a key transcriptional regulator for cancer and inflammation-related diseases [44, 45]. MiR-155-5p promotes the polarization of monocytes towards the M1 lineage, being in negative correlation with the suppressor of cytokine signaling 1 (SOCS1) expression [21, 22, 46]. Previous in vitro study on bovine CD14+ monocytes challenged with Staphylococcal enterotoxin B (SEB) demonstrated a decrease in miR-155-5p level, suggesting that *S. aureus* may induce immunosuppression to survive inside the host [47]. Conversely, this study showed the upregulation of miR-155-5p in mononuclear cells stimulated with GTB/ST8 secretome, consistently with the upregulation of the STAT1 expression. Both MiR-155-5p and STAT1 are regulated by positive feedback in response to inflammatory signals or infection [48]. MiR-155-5p modulates STAT1 expression suppressing SOCS1 expression in hepatoma cells and, thus, promoting the JAK/STAT signaling [49, 50].

The current study suffers from some limitations. Since no data have been previously reported on the ability of GTS/ST398 and GTB/ST8 to modulate bovine mononuclear cell response, the present investigation focused on the whole PBMC population. Further studies will investigate if and how the secretome produced by different *S. aureus* strains may promote macrophage and lymphocyte polarization toward different subpopulations. Moreover, the cells’ viability has been tested using the MTT test, while no information on death pathways, including pyroptosis, in which IL-1β plays a pivotal role [51], or necroptosis, efficiently promoted by *S. aureus* [52, 53], has been evaluated. Finally, in this work, we used the growth conditions previously applied for the proteomic characterization of the *S. aureus* secretome, carried out on the same strains, to ensure reproducibility [10]. Nevertheless, further adjustments of culture conditions might improve *S. aureus* growth that more closely resembles the in vivo situation during mastitis [54], and this should be considered in future research.

The present study demonstrated for the first time that the molecules secreted by *S. aureus* can modulate the immune response of bovine leukocytes in vitro, highlighting how secretomes from *S. aureus* strains with different epidemiological behaviors could elicit dramatically different responses in bovine PBMCs. The GTS/ST398 secretome led to significant losses in cell viability, while the GTB/ST8 secretome positively affected bovine PBMC viability. The immune response was also studied at a molecular level for GTB/ST8, revealing that the bacterial secretome can trigger the upregulation of genes involved in the Th1/M1 polarization. On the contrary, no effects could be observed in the expression of targets involved in the anti-inflammatory response. Further studies on *S. aureus-*secreted proteins will clarify whether stimulating secretomes isolated from different strains may differentially modulate the host immune cells’ response.

## Data Availability

The datasets used and/or analyzed during the current study are available from the corresponding author upon reasonable request.
